# Independent mechanisms recruit the cohesin loader protein NIPBL to sites of DNA damage

**DOI:** 10.1242/jcs.197236

**Published:** 2017-03-15

**Authors:** Christopher Bot, Annika Pfeiffer, Fosco Giordano, Dharani E. Manjeera, Nico P. Dantuma, Lena Ström

**Affiliations:** Karolinska Institutet, Department of Cell and Molecular Biology, Stockholm 171 77, Sweden

**Keywords:** Cohesin, DNA damage, DNA damage recruitment, Genome stability, Laser microirradiation, NIPBL

## Abstract

NIPBL is required to load the cohesin complex on to DNA. While the canonical role of cohesin is to couple replicated sister chromatids together until the onset of mitosis, it also promotes tolerance to DNA damage. Here, we show that NIPBL is recruited to DNA damage throughout the cell cycle via independent mechanisms, influenced by type of damage. First, the heterochromatin protein HP1γ (also known as CBX3) recruits NIPBL to DNA double-strand breaks (DSBs) through the corresponding HP1-binding motif within the N-terminus. By contrast, the C-terminal HEAT repeat domain is unable to recruit NIPBL to DSBs but independently targets NIPBL to laser microirradiation-induced DNA damage. Each mechanism is dependent on the RNF8 and RNF168 ubiquitylation pathway, while the recruitment of the HEAT repeat domain requires further ATM or ATR activity. Thus, NIPBL has evolved a sophisticated response to damaged DNA that is influenced by the form of damage, suggesting a highly dynamic role for NIPBL in maintaining genomic stability.

## Abstract (precis)

**Summary:** Accumulation of NIPBL, the cohesin loader, at different types of DNA lesions occurs through independent mechanisms. This finding enhances our understanding of the highly dynamic roles for NIPBL in genome stability.

## INTRODUCTION

The cohesin complex is fundamental to the maintenance of genomic integrity, and ensures proper segregation of the paired sister chromatids during mitosis. The core cohesin complex consists of four constituents, with Smc1 family proteins, Smc3 and RAD21 (Scc1 in budding yeast) forming a heterotrimeric ring structure, which then associates with either of the SA1 or SA2 stromal antigen proteins (STAG1 or STAG2; Scc3 in budding yeast) (Nasmyth and Haering, 2005). In mammalian cells, the initial association of cohesin with DNA occurs during telophase, and is facilitated by a separate ‘loader’ complex comprising of the NIPBL and MAU2 heterodimer (Scc2 and Scc4 in budding yeast) (Ciosk et al., 2000; Sumara et al., 2000; Watrin et al., 2006). Beyond the canonical role of cohesin in ensuring faithful chromosome segregation, it also promotes the correct repair of damaged DNA (Sjogren and Nasmyth, 2001; Watrin and Peters, 2009). Recently it has also been shown that the loss of the core RAD21 or the sororin accessory protein (also known as CDCA5) leads to aberrant non-homologous end-joining of distant DNA double-strand breaks (DSBs), resulting in large chromosomal rearrangements (Gelot et al., 2016). How cohesin participates in DNA repair is presently unclear, although it can be distinguished from its role in sister chromatid cohesion. For example, in response to DNA damage, cohesin is sumoylated by the MMS21 SUMO ligase (also known as NSMCE2) component of the cohesin-related SMC5–SMC6 complex (hereafter denoted SMC5/6). While this sumoylation is required for repair of DSBs by sister chromatid homologous recombination, it is dispensable for maintaining sister chromatid cohesion (Wu et al., 2012).

As well as post-translational modification of existing cohesin, additional cohesin complexes also appear to be recruited at sites of DNA damage. In budding yeast, analysis of regions flanking endonuclease-derived DSBs revealed a local enrichment of cohesin, in a manner dependent on the NIPBL–MAU2 loader complex (Strom et al., 2004; Unal et al., 2004). Early components of the DNA damage response (DDR) are also required for the accumulation of cohesin at DSBs, since in the absence of γH2AX and Mre11 (Mre11a in mammals; part of the MRN complex), this recruitment is abolished. However, the nature of cohesin recruitment to DNA damage in human cells remains controversial, and the purpose of loading additional cohesin in DNA repair is enigmatic. Consistent with studies in budding yeast, an enrichment of human cohesin could be detected at various genomic sites of endonuclease derived DSBs by ChIP-qPCR (Caron et al., 2012). Here, the cohesin recruitment was only moderate, but consistently detected throughout the cell cycle, and featured equal enrichment of the cohesin complex bound to either of the SA1 or SA2 associated factors. In addition, accumulation of both the NIPBL loader protein and cohesin could also be observed by fluorescence microscopy at I-PpoI endonuclease-derived DSB sites, which occur predominantly within the ribosomal DNA (Kong et al., 2014; Oka et al., 2011). However, at these I-PpoI sites, the accumulation of cohesin was only observed in S and G2 phases of the cell cycle, and was specifically enriched for the SA2 associated factor (Kong et al., 2014). The recruitment of NIPBL and cohesin–SA2 to DNA damage during the S and G2 phase of the cell cycle has also been observed upon inflicting DNA damage by laser microirradiation (Kim et al., 2002; Kong et al., 2014), although in another study using a laser optimised for DSB generation, no enrichment of cohesin was observed in immunofluorescence analyses (Bekker-Jensen et al., 2006), suggesting that other forms of DNA damage may also recruit cohesin. To better understand the basis for cohesin loading at sites of DNA damage in human cells, we have examined the recruitment of the human NIPBL cohesin loader protein to various types of damaged DNA. By exploiting stable cell lines inducibly expressing GFP-tagged NIPBL, we show that NIPBL contains at least two domains that can independently facilitate its recruitment to DNA lesions and that the type of DNA damage influences their relative contribution.

## RESULTS

### NIPBL and MAU2 are recruited to DNA damage

**Fig. 1.**
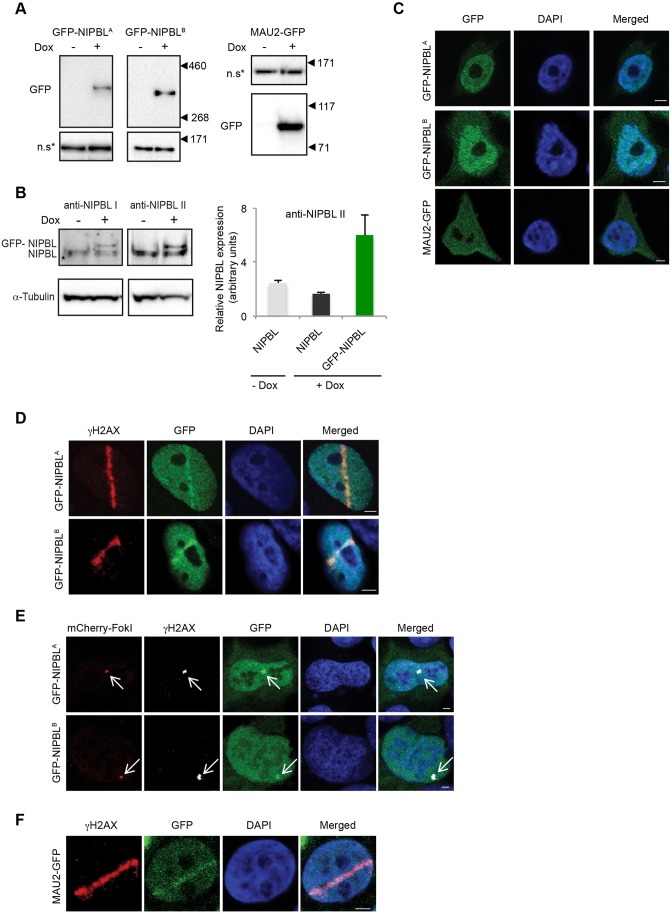
**NIPBL and MAU2 are recruited to sites of DNA damage.** (A) Ectopic gene expression in HEK293 cells stably expressing GFP fusions of NIPBL^A^, NIPBL^B^ or MAU2 was induced by doxycycline and detected after 48 h by immunoblotting with an anti-GFP antibody. Size marker positions are indicated, and a non-specific GFP antibody band (n.s*) illustrates gel loading. The expanded blot of the full-length NIPBL isoform A is also shown in Fig. 3B. (B) Western blots of endogenous NIPBL and GFP–NIPBL^A^ from the stable NIPBL^A^ cell line induced with doxycycline for 48 h. The expression level of ectopic GFP–NIPBL^A^, was quantified through comparison of the endogenous NIPBL and GFP–NIPBL bands, using two different anti-NIPBL antibodies (I and II), to their common loading control. These values were adjusted to compensate for GFP–NIPBL^A^ only being expressed in 18–20% of the cells as determined by FACS analysis (data not shown). The graph shows the mean± s.d. (*n*=3) band intensities of indicated proteins after compensation for the percentage of GFP-positive cells. (C) Confocal microscopy of fixed HEK293 interphase cells expressing NIPBL and MAU2 GFP fusion proteins. (D) HEK293 cells inducibly expressing either GFP–NIPBL isoform A or B were laser microirradiated, fixed after 30 min and immunolabeled for γH2AX. (E) FokI U2OS cells were transiently transfected with plasmids encoding either GFP–NIPBL isoform A or B. Activity of the FokI nuclease was induced at 19 h post transfection. Cells were immunostained for γH2AX and imaged by confocal microscopy. Arrows point at the LacO array. (F) HEK293 cells stably expressing MAU2–GFP were laser microirradiated and labeled as in D. Scale bars: 3 μm.

Higher eukaryotes express two protein isoforms of NIPBL (Strachan, 2005), and although the mammalian variants are derived from alternative splicing of the 3′ end of the gene, it is notable that fish, birds, and reptiles possess two discrete NIPBL gene paralogs (Ensemble database), suggestive of isoform-specific functions. Thus, we first set out to determine which human NIPBL variant is recruited to DNA damage. In humans, the canonical A-isoform (denoted NIPBL^A^) is a 316 kDa protein, while 3′ alternative splicing results in the slightly smaller 304 kDa B-isoform (denoted NIPBL^B^). We cloned the coding sequences for both NIPBL isoforms and fused each to GFP. Owing to the considerable size and low transfection efficiency of NIPBL, we opted to generate stable HEK293 cell lines regulated by the tetracycline repressor system to ensure that the transgenes were properly expressed. To examine MAU2 localization in relation to DNA damage, we also generated a MAU2–GFP fusion cell line. Western blot analysis confirmed proper full-length expression of the fusion proteins for each cell line (Fig. 1A). The expression level of ectopic GFP–NIPBL^A^, as shown on the western blot in Fig. 1B, was quantified through correlation of the GFP–NIPBL and endogenous NIPBL bands, using two different anti-NIPBL antibodies (Enervald et al., 2013; Zuin et al., 2014), to their common loading control, and then compensating for the fact that only 18–20% of the cells in the population are expressing GFP–NIPBL, as determined by FACS analysis (Fig. 1B; data not shown). The average level of GFP–NIPBL expression was found to be four times higher than that of endogenous NIPBL after 48 h in the presence of doxycycline. Fluorescence microscopy showed that the GFP–NIPBL fusions localized predominantly to the nucleus (Fig. 1C). The MAU2 fusion often appeared dispersed between the nucleus and the cytoplasm, as described previously for ectopic MAU2 (Seitan et al., 2006). Potentially this dispersal is due to the limited availability of physiological NIPBL that could be required to convey MAU2 to the nucleus, as *in silico* analyses failed to identify any high-probability nuclear localization signal sequences within MAU2.

To assess the response of the two NIPBL isoforms towards DNA damage, we first applied 365 nm UV-A laser microirradiation (Lukas et al., 2004) to each stable cell line. Both isoforms clearly accumulated at the resulting track lines, as defined by the DNA damage marker histone γH2AX (Fig. 1D). In parallel, we inflicted an alternative source of DNA damage by utilizing an engineered U2OS cell line in which DSBs can be enzymatically induced at an integrated LacO array by the FokI nuclease (Tang et al., 2013). Following transient transfection of the expression plasmids, we found that both isoforms of NIPBL accumulated at DSBs (Fig. 1E). Next, we investigated whether MAU2 was recruited to DNA damage. Laser microirradiation of the MAU2–GFP cell line resulted in the accumulation of MAU2 at DNA damage tracks (Fig. 1F), revealing that both components of the NIPBL–MAU2 heterodimer are recruited to damaged DNA. We were unable to assess localization of MAU2 to FokI-inflicted DSBs since transient overexpression of MAU2 only resulted in cytoplasmic protein aggregates.

### MAU2 does not function as a chromatin adapter for GFP-NIPBL at damaged DNA

**Fig. 2.**
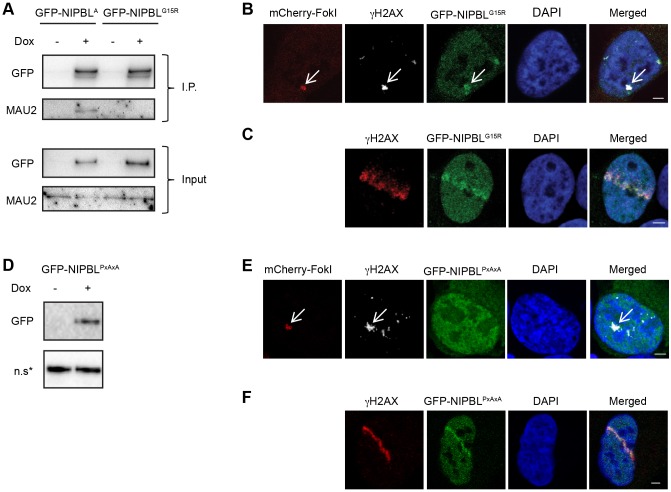
**GFP–NIPBL is recruited to DNA damage independently of MAU2, and HP1 mediates the recruitment of NIPBL only to DSBs.** (A) The expression of GFP fusions for either wild-type NIPBL isoform A (GFP–NIPBL^A^) or NIPBL isoform A featuring the G15R mutation (GFP–NIPBL^G15R^) were induced with doxycycline for 48 h. NIPBL was immunoprecipitated (I.P.) on GFP–Sepharose beads and then probed for associated native MAU2 protein by western blotting. (B) The FokI cells were transiently transfected with GFP–NIPBL^G15R^ and fixed 5 h after induction. Cells were imaged via confocal microscopy. (C) Following 48 h of induced GFP–NIPBL^G15R^ expression, cells were laser microirradiated, and labeled as in Fig. 1D. (D) A HEK293 stable cell line was generated for GFP–NIPBL incorporating the PxVxL to PxAxA double mutation within the HP1-binding motif (GFP–NIPBL^PxAxA^). (E) A representative confocal microscopy image of the FokI U2OS cell line transiently transfected with the GFP–NIPBL^PxAxA^ plasmid. (F) The GFP–NIPBL^PxAxA^ stable cell line was induced for 48 h, and then laser microirradiated, fixed after 30 min and immunolabeled for γH2AX. Arrows in B and E highlight the LacO array. Scale bars: 3 μm.

To explore the regulation of NIPBL in DNA repair further, we set out to determine how NIPBL is recruited to DNA damage. Since both NIPBL isoforms are recruited to DNA damage, the following experiments were based on the canonical NIPBL^A^ isoform. Interestingly, MAU2 is not required for cohesin loading *in vitro* (Murayama and Uhlmann, 2014), although it is essential for the *in vivo* loading of cohesin required for faithful chromosome segregation (Ciosk et al., 2000; Seitan et al., 2006; Watrin et al., 2006), and for effective DNA repair in budding yeast (Strom et al., 2004). While the function of MAU2 is currently unknown, it has recently been suggested that MAU2 may act *in vivo* as a chromatin adapter that targets NIPBL to specific chromosomal protein receptor sites (Chao et al., 2015). To explore whether this occurs in respect to damaged chromatin, we disrupted the MAU2-binding site of NIPBL and then examined the ability of NIPBL to accumulate at DNA damage. A single NIPBL missense mutation, derived from a Cornelia de Lange Syndrome (CdLS) patient, prevents a 300-amino-acid NIPBL fragment from binding MAU2 (Braunholz et al., 2012). Therefore, to precisely disrupt the NIPBL–MAU2 association without affecting overall NIPBL protein structure, we introduced this mutation (G15R) into full-length GFP–NIPBL, and constructed a stable cell line. Co-immunoprecipitation of native MAU2 from GFP–NIPBL versus GFP–NIPBL^G15R^ cell lines validated the disruption of MAU2 binding only towards the mutant protein (Fig. 2A). Therefore, the single G15R mutation is sufficient to disrupt the binding of MAU2 to full-length NIPBL in human cells. However, despite the de-coupling of MAU2 from GFP–NIPBL^G15R^, we still observed the accumulation of GFP–NIPBL^G15R^ at FokI-induced damage foci (Fig. 2B) and at laser damage tracks (Fig. 2C), suggesting that MAU2 is not absolutely required as a chromatin adapter for NIPBL at damaged DNA. Thus, moderately overexpressed ectopic full-length NIPBL (Fig. 1B) is recruited to damaged DNA independently of MAU2.

### Multiple protein domains recruit NIPBL to DNA damage

Heterochromatin protein 1 (HP1) represents another candidate for NIPBL recruitment to sites of DNA damage. Previously it was shown that the expression of a small NIPBL fragment featuring the HP1-binding domain generated a protein product that could recognise damaged DNA and that upon mutation of the HP1 motif from PxVxL to PxAxA, which abolished HP1 binding, this property was lost (Oka et al., 2011). We therefore investigated whether the same phenotype occurred when the identical mutation was introduced into full-length NIPBL and expressed in a stable cell line (Fig. 2D). We observed no enrichment of GFP–NIPBL^PxAxA^ at FokI nuclease-mediated DNA damage foci (Fig. 2E), a result consistent with the study performed using the protein fragment (Oka et al., 2011). Surprisingly, however, we found that the GFP–NIPBL^PxAxA^ still accumulated at laser damage tracks (Fig. 2F), which indicated the presence of a second HP1-independent recruitment mechanism for NIPBL.

**Fig. 3.**
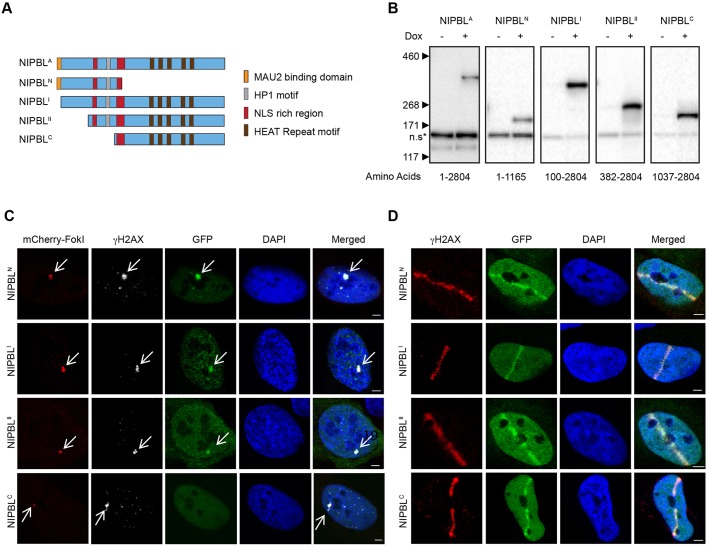
**Distinct domains mediate the recruitment of NIPBL to sites of DNA damage.** (A) An illustration showing notable predicted protein features for NIPBL, and the various protein truncations of NIPBL isoform A. (B) Western blots of protein lysates extracted from stably transformed HEK293 cells inducibly expressing each protein truncation fused to GFP. Expressed fusions are detected by an anti-GFP antibody 48 h after induction. Approximate positions of the protein size markers (in kDa) are indicated, and the GFP non-specific band (*) indicates the gel mobility of each truncation relative to one another. An expanded blot of full-length NIPBL isoform A taken from Fig. 1 is shown for comparison. (C) The FokI U2OS cell line was transiently transfected with plasmids for each truncation. The FokI nuclease was then induced, and 5 h later cells were fixed and immunostained for γH2AX. (D) Each NIPBL truncated cell line was induced for 48 h, laser microirradiated and then immunostained as standard (see also Fig. S1). Arrows in C and D highlight the LacO array. Scale bars: 3 μm.

To explore this further, we constructed a series of GFP–NIPBL truncations (Fig. 3A), and generated stable cell lines for expression of each fragment (Fig. 3B). Further truncation of the N-terminus was limited by the position of the central nuclear localization signal (NLS), which we delineated to be between amino acids 1037–1166, while milder C-terminal truncations resulted in unstable fusion proteins. In line with the recruitment pattern of the NIPBL^PxAxA^ mutant, only truncations possessing the HP1-binding motif were recruited to FokI damage foci. The minimal C-terminal NIPBL fragment (NIPBL^C^), lacking the HP1 domain, was never observed at FokI damage sites (Fig. 3C). In contrast, all truncated forms of NIPBL, including the minimal C-terminal fragment, accumulated at laser damage tracks (Fig. 3D). This suggested the presence of a second protein domain within the C-terminal part that separately recruits NIPBL to laser damage. Notably, a fragment consisting of the NLS-rich domain shared by both the minimal N-terminal and C-terminal fragments was not recruited to laser-based DNA damage, excluding the possibility that this domain facilitated the accrual of both fragments (Fig. S1A,B). It was also possible that the C-terminal-based mechanism could be isoform specific. We therefore transiently transfected the minimal C-terminal fragment of NIPBL^B^ and inflicted laser irradiation. We observed a clear recruitment of the B-variant to laser damage tracks, but, as expected, not to FokI nuclease sites (Fig. S1C,D). Taken together, this demonstrates that the C-terminal recruitment mechanism functions through the large domain containing the multiple HEAT repeat motif common to both isoforms, situated downstream of the NLS. Furthermore, this also reveals that NIPBL features separate DNA damage recruitment domains, where the NIPBL^PxAxA^ mutant phenotype was masked by the capacity of the HEAT repeat domain of NIPBL^C^ to independently target DNA damage.

**Fig. 4.**
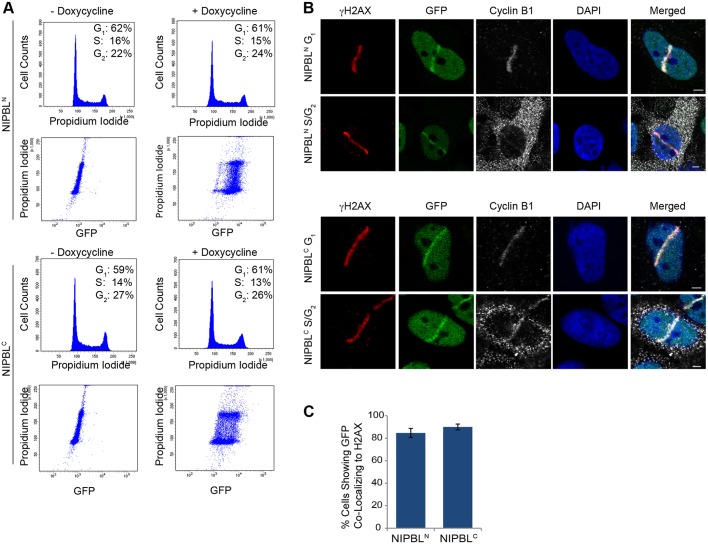
**NIPBL accumulates at laser-induced DNA damage throughout the cell cycle.** (A) FACS analysis of the cell cycle distribution and GFP expression of the NIPBL^N^ and NIPBL^C^ stable cell lines in the absence or presence of doxycycline for 48 h. The fraction of cells residing in each cell cycle phase based on propidium iodide incorporation is indicated. (B) The NIPBL^N^ and NIPBL^C^ stable cell-lines were induced with doxycycline for 48 h and then laser microirradiated as standard. Cells were immunolabeled for both γH2AX (Cy3), and cyclin B1 (Alexa Fluor 647). Cells positive for cyclin B1 were considered to be in either S or G_2_ phase. Cells lacking cyclin B1 commonly showed recruitment of either NIPBL^N^ or NIPBL^C^ to DNA damage tracks. Note the γH2AX signal from the red channel bleeds through into the weaker signal of cyclin B1 in the far-red channel. (C) Quantification of the number of cells displaying GFP-NIPBL recruitment to γH2AX lines in each group irrespective of cell cycle phase. Results are mean±s.d., *n*=2. Scale bars: 3 μm.

Since two distinct protein domains could recruit NIPBL to DNA laser damage, we exploited our stable cell lines expressing the smallest N-terminal fragment (NIPBL^N^) and the minimal C-terminal fragment (NIPBL^C^) to study each domain separately. Flow cytometry analysis showed that both NIPBL^N^ and NIPBL^C^ cell lines display normal cell cycle DNA content profiles, and that expression of each NIPBL fragment is consistent throughout the cell cycle (Fig. 4A). With our laser microirradiation system, we observed accumulation of NIPBL^N^ and NIPBL^C^ at γH2AX-labeled DNA damage tracks in cells expressing cyclin B1, an S- and G2-phase-specific marker, and in cells that did not display cyclin B1, indicating those cells that have not entered S phase (Fig. 4B). Indeed, the clear majority of cells targeted for laser damage display NIPBL^N^ and NIPBL^C^ recruitment to γH2AX-labeled damage lines, regardless of cell cycle stage (Fig. 4C).

### Independent mechanisms recruit NIPBL to DNA damage

**Fig. 5.**
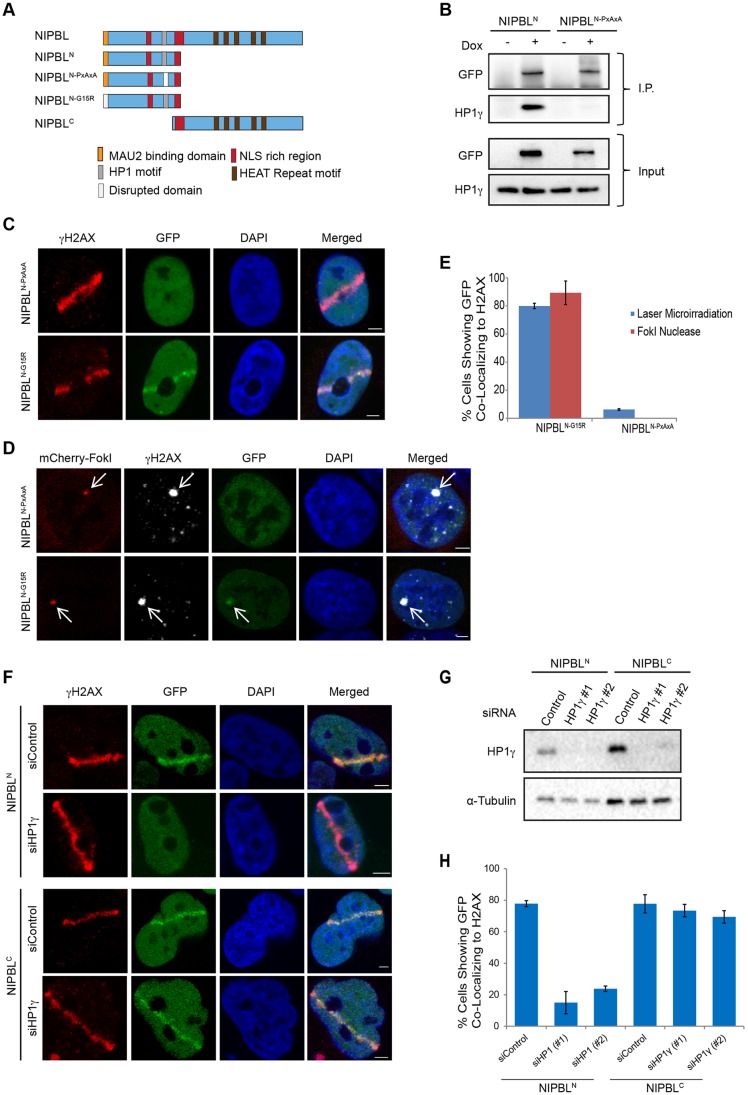
**The HP1 protein only recruits the N-terminal domain of NIPBL to DNA damage.** (A) Schematic of the mutations used to disrupt either MAU2 binding (G15R) or HP1 binding (PxAxA) in the C-terminally truncated form of NIPBL that can be stably expressed (NIPBL^N^), and the maximum truncation possible of the N-terminal (NIPBL^C^). (B) Disruption of HP1 binding to NIPBL^N^, via the PxAxA mutation, was demonstrated by immunoprecipitation (I.P.) of stable wild-type NIPBL^N^ or NIPBL^N-PxAxA^, with GFP–Sepharose beads and detection of co-immunoprecipitated HP1γ protein by western blotting. (C) NIPBL^N-G15R^ and NIPBL^N-PxAxA^ plasmids were transiently transfected into the HEK293/FRT/TO parental cell line. Expression was induced upon transfection with doxycycline, and 24 h later cells were microirradiated and then fixed as standard. (D) The FokI U2OS cell line was transiently transfected with NIPBL^N-G15R^ and NIPBL^N-PxAxA^ plasmids and assayed as described previously. (E) Quantification of the results from each FokI and laser microirradiation experiment. Results are mean±s.d., *n*=2. (F) The NIPBL^N^ and NIPBL^C^ stable cell lines were simultaneously transfected with the specified siRNA and induced with doxycycline for 48 h. Cells were microirradiated, then fixed as usual. A representative image from one transfection is shown; both HP1γ siRNAs behaved identically. (G) A western blot validating the knockdown efficiency of two different HP1γ siRNAs when transfected for 48 h in the NIPBL^N^ and NIPBL^C^ stable cell lines. Scrambled siRNA is used as a control. (H) Quantification of the experiments. Results are mean±s.d., *n*=2. Arrows in D highlight the LacO array. Scale bars: 3 μm.

With it being established that NIPBL^N^ and NIPBL^C^ would normally be recruited by laser microirradiation in HEK293 cells, we began to further dissect the underlying mechanisms. Our data suggest that the HP1-binding motif is required for recruiting NIPBL^N^ to laser microirradiation and FokI nuclease-based DNA damage, as reported for a smaller fragment which can recognize PpoI nuclease and UV-C-based microirradiation DNA damage (Oka et al., 2011). However, given the potential for the recruitment of NIPBL^C^ to mask the contribution of NIPBL^N^, it was also feasible that MAU2 could still influence the recruitment of the NIPBL^N^ fragment to laser damage. To formally examine this, we introduced the mutations that disrupted HP1 or MAU2 binding into the N-terminal domain (Fig. 5A), forming NIPBL^N-PxAxA^ and NIPBL^N-G15R^ expression plasmids. To ensure that the PxAxA mutation disrupted the interaction between NIPBL^N^ and HP1, we assessed HP1 binding to NIPBL by co-immunoprecipitation, which confirmed that HP1 did not interact with the truncated PxAxA mutant (Fig. 5B). As expected, NIPBL^N-G15R^ accumulated at laser damage sites, in an analogous manner to its full-length counterpart. However, the recruitment of NIPBL^N-PxAxA^ to damage lines was severely diminished (Fig. 5C,E). Similar results were obtained using the FokI nuclease system, where NIPBL^N-G15R^ accumulated at damage foci, while the recruitment of NIPBL^N-PxAxA^ to nuclease sites was never observed (Fig. 5D,E). Thus, MAU2 likely plays no role in assisting the HP1-mediated recruitment mechanism. An absence of a second HP1-binding motif in the C-terminal indicated that two independent mechanisms recruit NIPBL to DNA damage: the first via the N-terminal HP1 motif, and the second by the C-terminal HEAT repeat domain. Thus, depletion of HP1 should only inhibit the accumulation of NIPBL^N^ and not impede the recruitment of NIPBL^C^. Of the three mammalian HP1 isoforms (HP1α, β and γ; also known as CBX5, CBX1 and CBX3, respectively), only HP1γ appears to retain the HP1-binding NIPBL fragment at DNA damage (Oka et al., 2011). We therefore depleted HP1γ in our stable cell lines and compared the recruitment of NIPBL^N^ and NIPBL^C^. In agreement with our hypothesis, upon RNAi-mediated depletion of HP1γ, the recruitment of NIPBL^N^ was severely diminished, while the recruitment of NIPBL^C^ remained unaffected (Fig. 5F–H). Therefore, two separate mechanisms are involved in recruiting NIPBL to DNA damage.

**Fig. 6.**
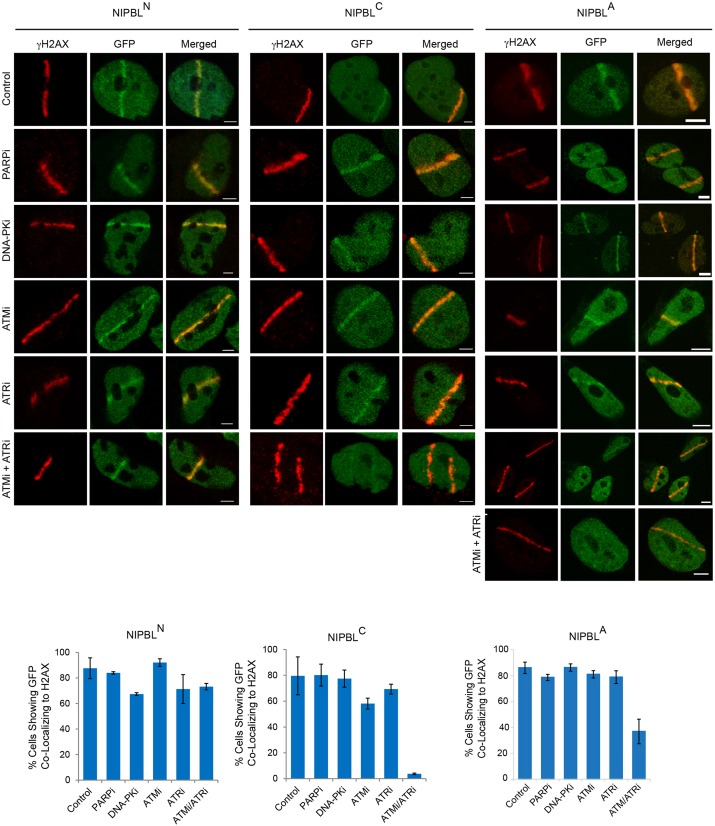
**Recruitment of the C-terminal domain of NIPBL to DNA damage is mediated by ATM or ATR activity.** HEK293 cells stably expressing either NIPBL^N^, NIPBL^C^ or full-length NIPBL^A^ were induced for 48 h, and treated with 10 μM inhibitor (PARPi, KU-0058948; DNA-PKi, Wortmannin; ATMi, KU-60019; ATRi, AZD6738) for 1 h prior to laser microirradiation. All inhibitors were functionally validated to ensure expected activity (Fig. S3). Following laser damage, the cells remained in medium containing the specified inhibitor for a further 30 min, before fixation and immunostaining for γH2AX. For dual ATM and ATR inhibition (denoted ATMi+ATRi or ATM/ATRi), each inhibitor was added at 7.5 μM to maintain DMSO levels below 0.1%. For NIPBL^A^, representative images for both cells with NIPBL recruitment and without are shown. The fluorescence of the red channel was enhanced when necessary for visualization purposes to compensate for a reduced γH2AX signal resulting from DNA-PK, ATM or ATR inhibition. Quantification of the experiments is shown below. Results are mean±s.d., *n*=2. Scale bars: 5 μm.

We then set out to determine factors that are required for the recruitment of NIPBL^C^ to laser damage. Protein sequence analysis indicated that while the NIPBL^N^ region featuring the HP1-binding motif is mostly absent from lower eukaryotic orthologs, there appears to be a degree of conservation of NIPBL^C^ throughout the Eukaryota. Therefore, since the loading of cohesin at sites of DNA damage appears to be evolutionarily conserved between yeast and humans, and is abolished by deletion of ATM and ATR in *Saccharomyces* *cerevisiae* (Kim et al., 2002; Tittel-Elmer et al., 2012; Unal et al., 2004), we investigated whether these factors would influence the recruitment of NIPBL^C^. Highly specific inhibitors for ATM (KU-60019) and ATR (AZD6738) can be used to distinguish the activity of these kinases from the many other members of the phosphoinositide 3-kinase (PI3K) family (Golding et al., 2009; Vendetti et al., 2015). The effectiveness of the inhibitors was validated by western blotting for typical biomarkers (Fig. S2). Interestingly, while chemical inhibition of ATM or ATR did not prevent NIPBL^N^ or NIPBL^C^ from being recruited to laser microirradiation, dual inhibition of ATM and ATR abolished the recruitment of NIPBL^C^ almost completely (Fig. 6, left and central panels, respectively). In contrast, the recruitment of NIPBL^N^ remained following combined ATM and ATR inhibition, again confirming that independent mechanisms recruit NIPBL to DNA damage. That inhibition of ATM alone had no effect on the recruitment of the NIPBL cohesin loader protein is also consistent with the observation that cohesin is recruited to sites of laser microirradiation in ATM-deficient A-T cells (Kim et al., 2002). We then tested two DNA damage signaling components absent from *S. cerevisiae*: the DNA damage sensitive kinase DNA-PK and the poly(ADP-ribose) polymerase PARP1. Wortmannin selectively blocks DNA-PK activity, although at higher concentrations it can also inhibit ATM (but not ATR) (Sarkaria et al., 1998), while KU0058948 inhibits PARP1 activity (Fig. S2). We found that neither inhibition of DNA-PK nor PARP1 prevented NIPBL^N^ or NIPBL^C^ from being recruited to DNA damage. We then investigated whether the combined inhibition of ATM and ATR would influence the recruitment of full-length NIPBL to laser damage. We found that the recruitment was reduced, but not completely abolished, with 40% of cells displaying NIPBL accumulation at laser tracks in the presence of both inhibitors. As for the N- and C-terminal NIPBL fragments, PARP or DNA-PK inhibition had no effect on recruitment of full-length NIPBL (Fig. 6, right panel). Thus, analogous to the requirements for cohesin loading in budding yeast, redundant ATM or ATR signaling influences the recruitment of the NIPBL protein to DNA damage.

### Both DNA damage recruitment mechanisms require RNF8 and RNF168 ubiquitin ligases

**Fig. 7.**
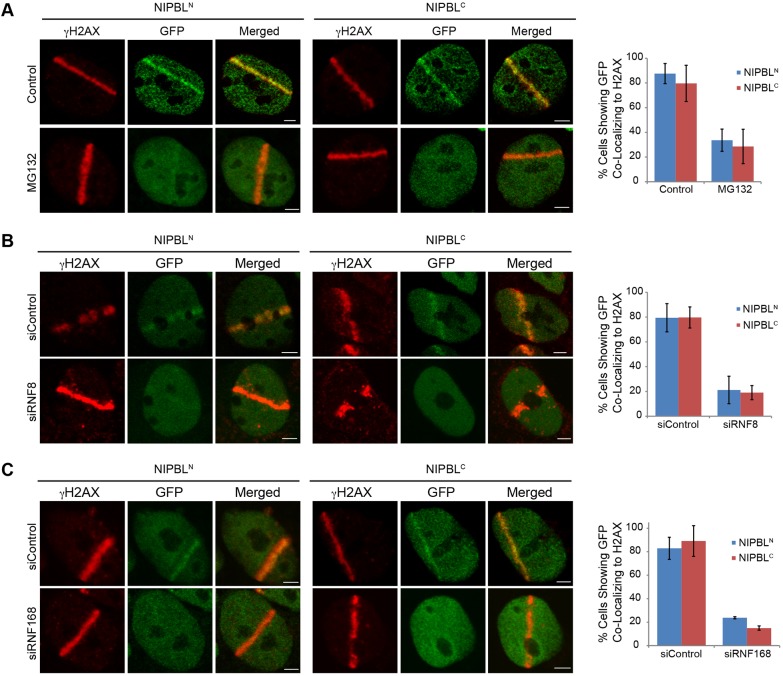
**RNF8, RNF168 and ubiquitin signaling are required for NIPBL recruitment to DNA damage.** (A) NIPBL^N^ or NIPBL^C^ cells were induced for 48 h, and treated with 10 μM MG132 for 1 h, then laser-microirradiated, and fixed after 30 min. (B) Cells were depleted of RNF8 by siRNA treatment for 48 h (siRNF8), then exposed to laser microirradiation, and fixed after 30 min. (C) Cells were depleted of RNF168 for 48 h, treated with laser micro-irradiation, and fixed after 30 min. Quantifications of all experiments are shown, results are mean±s.d., *n*=2 (see also Fig. S2). Scale bars: 3 μm.

Previously it has been shown that the recruitment of full-length NIPBL to DNA damage required the presence of the RNF168 ubiquitin ligase (Oka et al., 2011), implying that RNF168 influences both DNA damage recruitment mechanisms. RNF168 is usually associated with the DNA damage-dependent RNF8 and RNF168 signaling cascade, whereby the sensor protein MDC1 first recruits RNF8 to ubiquitylated histone H1 (Thorslund et al., 2015), which consequently recruits RNF168, and leads to polyubiquitylated histone H2A (Doil et al., 2009; Stewart et al., 2009). Polyubiquitylated H2A then recruits downstream factors including 53BP1, BRCA1 and intriguingly the cohesin-related SMC5/6 complex (Huen et al., 2007; Kolas et al., 2007; Mailand et al., 2007; Raschle et al., 2015). However, RNF168 can also act independently from RNF8 to directly ubiquitylate 53BP1 (Bohgaki et al., 2013). We therefore set out to further examine the role of ubiquitin in the recruitment of NIPBL to DNA damage. The nuclear pool of ubiquitin available for DNA damage signaling can be reduced via perturbation of proteasome activity with compound MG132 (Dantuma et al., 2006; Mailand et al., 2007). With MG132 pre-treated cells, we observed a discernible effect on the recruitment of NIPBL^N^ and NIPBL^C^ (Fig. 7A), supporting a role for ubiquitin in the recruitment of both NIPBL fragments to DNA damage. We therefore examined a possible role for the ubiquitin ligases RNF8 and RNF168, which are critical for the induction of DNA damage-induced ubiquitylation at sites of DNA damage. Following depletion of RNF8 and RNF168 (Fig. S3A), we observed that NIPBL^N^ and NIPBL^C^ no longer accumulated at laser microirradiation-based DNA damage (Fig. 7B,C), indicating that DNA damage-induced ubiquitylation promotes the recruitment of NIPBL. Interestingly, this feature is shared by each of the DNA damage recruitment mechanisms. It has previously been shown that the formation of γH2AX and the recruitment of 53BP1 to DNA damage can occur in the absence of ATM due to the functional redundancy of ATM with DNA-PK (Stiff et al., 2004). Consistent with a role for DNA-PK in activating the RNF168 pathway, we still observed the recruitment of RNF168 to DNA damage when both ATM and ATR were inhibited (Fig. S3B). Therefore the contribution of RNF8 and RNF168 in recruiting NIPBL^C^ to DNA damage is most likely a distinct aspect of the recruitment pathway from the requirement for ATM or ATR activity.

Since the SMC5/6 complex is also recruited to laser-based DNA damage via the RNF8 and RNF168 cascade, and does not form foci at DSBs (Potts and Yu, 2007), similar to NIPBL^C^, it was possible that the recruitment mechanisms for NIPBL^C^ and SMC5/6 are related. As RAD18 acts as an adapter between RNF168-mediated H2A ubiquitin chains and SMC5/6 recruitment (Raschle et al., 2015), we depleted RAD18 in our DNA damage assays. However, both NIPBL^N^ and NIPBL^C^ were recruited to laser-induced DNA damage in the absence of RAD18 (Fig. S4A–C), demonstrating that, in response to laser microirradiation, NIPBL and SMC5/6 are recruited by distinct branches of the RNF8 and RNF168 cascade. Enticingly, the same recent proteomics screen that identified the recruitment of SMC5/6 to psoralen-induced DNA interstrand cross-links (ICLs) also revealed both NIPBL and MAU2 as proteins that accumulate at these lesions (Raschle et al., 2015). This prompted us to investigate whether psoralen-induced ICLs would lead to the recruitment of NIPBL^C^. The pre-sensitization of cells with BrdU prior to laser microirradiation not only generates DSBs, but also causes the formation of base lesions and various photo-adducts (Reynolds et al., 2013). To promote the formation of ICLs, we instead pre-sensitized cells with trimethylpsoralen (TMP), which predominantly forms ICLs via activation upon UV exposure (Huang et al., 2013). In response to UV laser microirradiation, we observed an accumulation of NIPBL^C^ at γH2AX damage tracks, suggesting that NIPBL^C^ is recruited to ICL damage (Fig. S4D). In contrast, NIPBL^C^ was not observed at sites of local UV damage directly (Fig. S4E), while it did accumulate at DNA damage in a BrdU pre-sensitized cell-line deficient for the UV damage sensor XPC (XP4PA cells) (Fig. S3F). Taken together, this indicates that NIPBL^C^ is recruited to psoralen induced ICLs in a manner dependent on RNF8- and RNF168-mediated ubiquitylation, as well as ATM and ATR activity.

## DISCUSSION

**Fig. 8.**
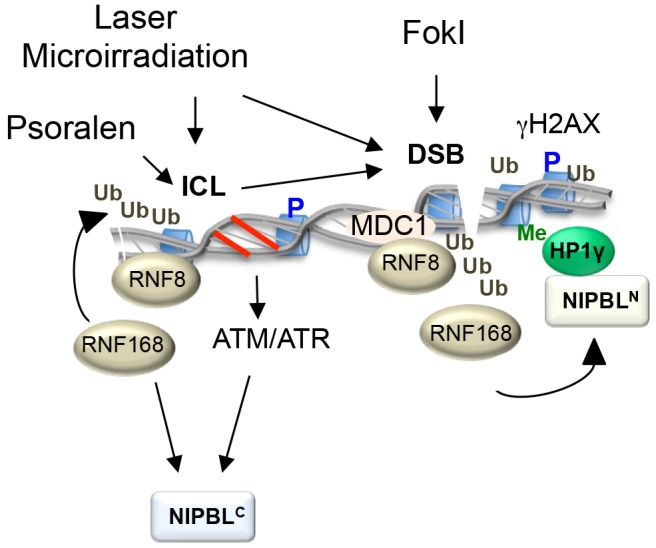
**A simplified model for recruitment of NIPBL to different types of DNA damage.** Illustrated is a summary of the factors found in this study to be important for the recruitment of NIPBL to different types of DNA damage via two independent pathways. NIPBL is recruited to DNA damage via interaction with HP1γ at the N-terminus, and via the ATM and ATR pathway through the C-terminal HEAT-repeat-rich domain. Upon induction of DNA damage via UV microirradiation (laser damage) different types of damaged lesions are induced, such as DSBs and ICLs. Recruitment of NIPBL to laser tracks is therefore seen both in the absence of HP1, and ATM and ATR, since the two independent pathways demonstrated here, act in a compensatory manner. FokI, by contrast, only induces DSBs, and recruitment to those is strictly dependent on HP1γ. A C-terminal fragment of NIPBL lacking the HP1-binding motif is therefore not recruited. Both pathways depend on ubiquitin and the RNF8 and RNF168 pathway. Me, methylation; Ub, ubiquitin; P, phosphorylation.

To facilitate the study of human NIPBL, we have developed stable cell lines for the inducible expression of full-length GFP–NIPBL fusion proteins. By describing two separate DDR pathways for NIPBL, we have demonstrated that independent mechanisms recruit NIPBL to sites of DNA damage, which can be distinguished by the type of inflicted DNA damage, summarized in Fig. 8. The first mechanism can target NIPBL to DSBs generated by endonucleases tethered to a repetitive LacO array, and requires an intact HP1-binding motif within NIPBL as well as the presence of HP1γ, RNF8 and RNF168. This suggests that the recruitment of NIPBL to DSBs is directly mediated via HP1γ acting as a chromatin adapter, whereby the chromodomain of HP1γ binds tri-methylated H3K9 (H3K9me_3_) and the opposing chromoshadow domain binds the PxVxL motif of NIPBL. However, as H3K9me_3_ and HP1γ are pre-existing epigenetic features of undamaged chromatin, RNF8- and RNF168-mediated ubiquitylation of chromatin could form the specific epigenetic marks that allow NIPBL to recognize DSBs, leading to NIPBL interacting with both H3K9me_3_–HP1γ and ubiquitylated chromatin in combination. This type of bivalent interaction would be analogous to the retention of 53BP1 by di-methylated H4K20 (H4K20me_2_) and RNF168-mediated H2A K15 ubiquitylation (Fradet-Turcotte et al., 2013), and may precisely define the chromatin locus that recruits NIPBL in response to a DSB. Alternatively, RNF8 and RNF168 activity may lead to a change in the local chromatin structure, facilitating the access of a pre-assembled HP1γ–NIPBL complex directly to H3K9me_3_ sites. Intriguingly, we did not observe NIPBL at microirradiation-induced DSB foci (C.B., unpublished data, Oka et al., 2011), which predominantly occur at sites of euchromatin (Cowell et al., 2007). This raises the possibility that the HP1-mediated recruitment of NIPBL is targeted to H3K9me_3_ regions more typical of heterochromatic DNA. In contrast, the evolutionarily conserved HEAT repeat domain of NIPBL was not observed at DSBs, and the accumulation at sites of laser microirradiation did not require HP1γ. Instead the laser damage recruitment of the HEAT repeat domain was dependent on RNF8 and RNF168, as well as ATM and ATR activity. Interestingly, the contribution of ATM and ATR activity appeared to be a separate aspect of the recruitment pathway from the RNF8 and RNF168 signaling cascade. This indicates that ubiquitylation of chromatin is not sufficient for the recruitment of NIPBL via the HEAT repeat domain, mirroring the ubiquitylation-dependent DSB response of NIPBL coupled to HP1γ. Thus, another bivalent mechanism may regulate the recruitment of NIPBL^C^ to DNA damage. Alternatively, the additional requirement for ATM and ATR activity may reflect the different type of damaged DNA that recruits NIPBL^C^, where potentially ATM and ATR activity is required to properly elicit the response to ICLs prior to NIPBL recruitment.

Following the recruitment of NIPBL to various types of DNA damage, it remains to be determined for each mechanism, whether NIPBL loads additional cohesin at sites of damaged DNA, re-locates existing chromatin-bound cohesin to damage regions, or even has a novel role at damaged DNA that is distinct from cohesin loading. However, our findings may help explain the apparent discrepancies between the relatively low levels of cohesin that accumulates adjacent to single nuclease generated DSB lesions randomly dispersed throughout the genome (Caron et al., 2012), and the greater enrichment of cohesin–SA2 at nuclease-derived DSB sites concentrated within the ribosomal DNA. The accumulation of cohesin at the ribosomal DNA region may reflect a concentrated recruitment of NIPBL to DSBs within these domains via the HP1γ and RNF8–RNF168 DDR pathway, since many inactive ribosomal genes are embedded within heterochromatin enriched for H3K9me_3_ (Grummt and Pikaard, 2003). In addition, the significant accumulation of cohesin to DNA damage induced by the 532 nm ND:YAG laser (Kong et al., 2014) may reflect the recruitment of NIPBL via the HEAT repeat domain to alternative forms of DNA damage that are generated by this type of laser, potentially recruiting cohesin to DNA interstrand cross-links. Indeed, if only moderate levels of cohesin are typically recruited to random genomic DSBs, as indicated by ChIP-qPCR (Caron et al., 2012), this may also explain the apparent lack of cohesin accumulation observed via the less-sensitive immuno-fluorescence analysis when using a laser set-up optimized specifically for the generation of DSBs (Bekker-Jensen et al., 2006).

Our study raises a fundamental question: why should a protein have multiple mechanisms for DNA damage recruitment? Parallel DNA damage recruitment processes are now emerging for a selection of DDR proteins including 53BP1, the SLX4 nuclease scaffold protein and the CHD4 chromatin remodeler (Dantuma and van Attikum, 2016). Parallel recruitment pathways may provide a higher degree of specificity for the DDR in terms of both the timing and the precise sites of recruitment of individual proteins. It may also allow the DNA damage response to be tailored towards the type of inflicted DNA damage. For example, SLX4 appears differentially recruited to DNA replication intermediates via SUMO binding, and to psoralen-induced ICLs via ubiquitin binding (Lachaud et al., 2014; Ouyang et al., 2015). This modularity is reminiscent of NIPBL, whereby recruitment to DSBs is mediated via HP1γ, while recruitment to ICLs can be mediated via the C-terminal HEAT repeat domain. Thus, it is tempting to speculate that the multiple DDR mechanisms of NIPBL regulate the specific sites and timing of cohesin loading in relation to the type of DNA damage that requires resolving. Appreciating that NIPBL can be recruited to alternative forms of DNA lesions via independent mechanisms represents a significant step forward in our understanding of the highly dynamic roles that NIPBL performs in maintaining genomic stability.

## MATERIALS AND METHODS

### Plasmids

To leave the NIPBL–MAU2 interaction unperturbed, a flexible amino acid spacer of 3×(GGGGS) was inserted between the N-terminal eGFP tag and NIPBL. The expression vector pCDNA5/FRT/TO (ThermoFisher) was modified to delete an internal *Mfe*I restriction site, while eGFP(ΔSTOP)-3×(GGGGS) was PCR amplified (primers GFP-NF/R; Table S1) and inserted into the *Afl*II and *Kpn*I sites. For RT-PCR, RNA was extracted with an RNeasy Mini-Kit (Qiagen), while cDNA was generated with a High Capacity cDNA Kit (ThermoFisher). The NIPBL isoform A was amplified from cDNA in component parts or synthesized [N-terminal (base pairs 1–925, primers NIPBL-NF/R), central (base pairs 919–4321, Blue Heron Biotechnology) and C-terminal (base pairs 4316–8415, primers NIPBL-CF/R)], and sequentially assembled via *Kpn*I, *Xho*I, *Pst*I, *Sal*I and *Not*I restriction sites. The C-terminal of NIPBL isoform B was cloned from cDNA (base pairs 7791–8097, primers NIPBL-BF/R), and inserted into NIPBL isoform A with *Xba*I/*Not*I. Both complete isoforms were transferred into pCDNA5/FRT/TO/GFP with *KpnI*/*Apa*I to make GFP–NIPBL^A^ and GFP–NIPBL^B^. Full-length MAU2^ΔSTOP^ was cloned from cDNA (primers MAU2-F/R) and inserted into pCDNA5/FRT/TO via *Hind*III/*Not*I; eGFP was amplified (primers GFP-CF/R) and fused to the C-terminal via *Not*I/*Apa*I. Gene truncations were created by PCR amplification and cloned into GFP–NIPBL^A^: NIPBL^I^ (base pairs 300–8415; primers NIPBL-F1/R1; cloned with *Kpn*I/*Xho*I), NIPBL^II^ (base pairs 1146–8415; primers NIPBL-F2/NIPBL-CR; cloned with *Kpn*I/*Mfe*I), NIPBL^C^ (base pairs 3111–8415; primers NIPBL-F3/NIPBL-CR; cloned with *Kpn*I/*Mfe*I), NIPBL^N^ (base pairs 1–3498; primers NIPBL-NF/NIPBL-R1; cloned with *Kpn*I/*Apa*I), and NIPBL^NLS^ (primers NIPBL-F3/ NIPBL-R1). For the B-isoform truncation, the variable region from GFP–NIPBL^B^ was sub-cloned to NIPBL^C^ (*Mfe*I/*Apa*I), to form NIPBL^C-B^. Site-directed mutagenesis was performed with the QuikChange II Site-Directed Mutagenesis Kit (Agilent) to give NIPBL^G15R^ (primers G15R-F/R) and NIPBL^PxAxA^ (primers PxAxA-F/R). The G15R (*Kpn*I/*Xho*I) and PxAxA mutants (*Xho*I/*Spe*I) were sub-cloned into NIPBL^N^ to make NIPBL^N-G15R^ and NIPBL^N-PxAxA^, respectively. All constructs were verified by sequencing. The DDB2-mCherry plasmid has been described previously (Alekseev et al., 2008). All primer sequences can be found in Table S1.

### Cell culture, transfections, and inhibitors

All cell lines, recently authenticated and tested for contamination, were cultured in Dulbecco's modified Eagle's medium (DMEM; Sigma), supplemented with 10% fetal calf serum (FCS). Stable transfection of pCDNA5/FRT/TO plasmids with Lipofectamine 2000 into HEK293 Flp-In™ T-REx™ (ThermoFisher), and selection of clonal cell lines was performed according to manufacturer's instructions. Expression of pCDNA5/FRT/TO derivatives in HEK293 Flp-In™ T-REx™ cells was induced with 1 μg ml^−1^ doxycycline (Sigma) for either 24 h (transient transfection) or 48 h (stable cell lines). FokI U2OS cells expressing ER–mCherry–LacR–FokI (Tang et al., 2013) were transfected for 19 h, followed by induction of FokI nuclease activity for 5 h with 1 µM 4-OHT and 1 µM Shield-1 (Clontech), before fixation. Prior to laser microirradiation, HEK293 cells were pre-sensitized with 10 µM BrdU for 24 h, or 5 µM trimethylpsoralen for 30 min. siRNA treatment was performed identically for either western blotting or laser micro-irradiation: 1×10^5^ cells were plated overnight, then transfected with 30 nM siRNA (Table S1) using Lipofectamine RNAiMAX reagent (ThermoFisher) for 48 h, prior to downstream experimentation. For chemical inhibition of ATM (KU-60019, Selleckchem), DNA-PK (Wortmannin, Sigma), PARP1 (KU-0058948, Toronto research chemicals), ATR (AZD6738), and the proteasome (MG132) cells were pre-treated with 10 μM inhibitor for 1 h (Farmer et al., 2005; Golding et al., 2009; Sarkaria et al., 1998; Vendetti et al., 2015). Dual ATM and ATR inhibition was performed with 7.5 μM of each inhibitor for 1 h. Each inhibitor was functionally validated by using western blotting – activation of each inhibitor target and lack of activation in the presence of the respective inhibitor (10 μM) was detected after induction of DNA damage for 1 h, using either bleomycin sulfate (10 μg/ml, Enzo Life Sciences) or hydroxyurea (2 mM) (see Fig. S2).

### Immunoprecipitation and immunoblotting

For co-immunoprecipitation of NIPBL and MAU2, cells were lysed in 0.5% NP-40, 150 mM NaCl, 50 mM Tris-HCl pH 8, 1 mM DTT, 1 mM PMSF and EDTA-free protease inhibitor (Roche), with the addition of 10 U of DNAseI (Thermo Scientific). After lysis, whole-cell extracts were centrifuged and the supernatant mixed with anti-GFP–Sepharose beads (ab69314, Abcam) overnight at 4°C, then washed six times in wash buffer (0.1% NP-40, 150 mM NaCl and 50 mM Tris-HCl pH 8). Proteins were released by heating to 80°C in buffer containing 50 mM HEPES and 1% SDS. For co-immunoprecipitation of NIPBL and HP1γ, cells were treated once with sucrose buffer [0.32 M sucrose, 3 mM CaCl_2_, 2 mM MgOAc, 0.1 mM EDTA, 1 mM DTT, 1 mM PMSF, EDTA-free protease inhibitor and the PhosSTOP phosphatase inhibitor (Roche)], and then a second time with sucrose buffer including 0.5% NP40. After centrifugation (1100 ***g*** for 10 min), the pellet was resuspended in nuclear lysis buffer (50 mM Hepes, 3 mM MgCl_2_, 300 mM NaCl plus proteinase and phosphatase inhibitors as before). DNA was removed by addition of benzonase. The nuclear extracts were incubated with Protein A beads coupled to anti-GFP antibody (Abcam) overnight at 4°C. Beads were then washed five times (1% NP40, 150 mM NaCl and 50 mM Tris-HCl pH 8). Finally nuclear extracts were eluted (50 mM Hepes, 1% SDS) at 65°C for 15 min and boiled in sample buffer before loading on SDS-PAGE gels for western blotting. For analysis of fusion proteins, inhibitor targets and siRNA experiments, whole-cell extracts were prepared in 0.1% SDS, 5 mM MgCl_2_ and 10 mM Tris-HCl (pH 8.2) lysis buffer, with protease inhibitors. For detection of kinase activity during validation of inhibitors, a phosphatase inhibitor was included (PhoSTOP, Roche). NIPBL was resolved by SDS-PAGE using 3–8% Tris-acetate gels (Bio-Rad), transferred onto PVDF membranes (Millipore) and detected via an anti-GFP antibody. Effects of inhibitors was analyzed on 4–12% Bis-Tris gels (Novex, Invitrogen).

### Microscopy

For live-cell microirradiation, a pulsed nitrogen laser (Photonic Instruments) was coupled to a Leica DMI 6000B microscope via the Leica ×40 HCX PL APO 1.25–0.75 oil-immersion objective (Acs et al., 2011). Laser output (20 Hz, 364 nm) was set to 11% (BrdU pre-treatment) or 4% (TMP pre-treatment) to inflict DNA damage against cells held in a 37°C chamber. Approximately 25 cells were damaged within a 5 min period, followed by a 25 min recovery time prior to fixation. Each experiment consisted of two replicates, and was repeated two or three times. All cells were fixed in 4% paraformaldehyde for 15 min. For localized UV damage, cells were UV irradiated through a polycarbonate mask (Millipore) with pores of 5 µm with doses of 25, 50 or 75 J/m^2^. For immunofluorescence labeling, cells were permeabilized in PBS containing 0.25% Triton X-100 for 5 min, incubated with primary antibody diluted in 3% BSA (Sigma) overnight at 4°C, followed by secondary antibody in 3% BSA at 37°C for 45 min, and mounted with ProLong Gold antifade with DAPI (Thermofisher). Images were acquired with a Zeiss LSM 510 META confocal microscope.

### Antibodies

The primary antibodies against the following proteins were used: GFP at 1:3000 (Abcam, ab290, ab69314), NIPBL I at 1:1000 (Enervald et al., 2013), NIPBL II at 1:500 (Santa Cruz, sc-374625) (Zuin et al., 2014), α-tubulin at 1:1000 (Sigma, T9026), γH2AX at 1:1000 (Millipore, 05-636), MAU2 at 1:200 (Visnes et al., 2014), HP1γ at 1:1000 (Millipore, 05-690 and Abcam, ab10480), RAD18 at 1:2500 (Abcam, AB57447), cyclin B1 at 1:400 (Santa Cruz Biotechnology, SC-245), RNF8 at 1:200 (Santa Cruz Biotechnology, SC-271462), RNF168 at 1:1000 (Millipore, ABE367), KAP1 at 1:1000 (Nordic Biosite, A300-274A), KAP1 phosphorylated at S824 at 1:1000 (Nordic Biosite, A300-767A), β-actin at 1:1000 (Abcam, ab8224), Chk1 at 1:1000 (Cell Signaling Technology, #2360), Chk1 phosphorylated at S345 at 1:1000 (Cell Signaling Technology, #2348), DNA-PKcs at 1:1000 (Thermo Fisher, MA5-13404), DNA-PKcs phosphorylated at S2056 at 1:1000 (Abcam, ab18192), poly(ADP-ribose) at 1:1000 (Enzo Life Sciences, 10H). All antibodies were previously validated by us or the respective manufacturer.

### Flow cytometry

Asynchronously growing cells either untreated or treated with 1 μg ml^−1^ doxycycline for 48 h were washed in ice-cold PBS, fixed in 70% ethanol at 4°C, and subsequently incubated with 40 µg/ml RNase A (Sigma-Aldrich) and 20 µg/ml propidium iodide (Sigma-Aldrich) at 37°C for 30 min. Analysis was performed using a BD FACSCANTO II (BD Biosciences). To enrich for cells expressing GFP–NIPBL^A^ for analysis of the effect of inhibitors on recruitment of full-length NIPBL to laser-induced damage sites, GFP-positive cells were sorted according to intensity of the FITC signal using a FACS Aria (BD Biosciences), 40 h after addition of doxycycline. The cells were then cultured for 24 h in the presence of doxycycline before treatment with inhibitors and exposure to laser damage as described above.
